# Is ^68^Ga-DOTA-FAPI a new arrow in the quiver of dose painting in radiation dose planning in head and neck cancers?

**DOI:** 10.1007/s00259-020-04895-8

**Published:** 2020-06-02

**Authors:** Patrick Conen, Felix M. Mottaghy

**Affiliations:** 1 Department of Nuclear Medicine, University Hospital RWTH Aachen University, Pauwelsstr. 30, 52074 Aachen, Germany; 2 Center of Integrated Oncology (CIO), Universities of Aachen, Bonn, Cologne, and Duesseldorf, Cologne, Germany; 3 Department of Radiology and Nuclear Medicine, Maastricht University Medical Center (MUMC+), P. Debeylaan 25, 6229 HX Maastricht, P.O. Box 5800, 6202 AZ Maastricht, The Netherlands

The majority of head and neck cancers is of epithelial origin with squamous cell carcinoma being the most common one [1]. Radiotherapy or combined radiochemotherapy is the main therapeutic option implemented as adjuvant or neoadjuvant approach. In recent years, sophisticated radiation dose planning using morphological and functional imaging modalities and the combination has evolved and has been proposed as a base for individualized radiotherapy planning [2]. However, until now, the clinical standard diagnostic tools to calculate the gross tumour volume (GTV) are computed tomography (CT) and magnetic resonance tomography imaging (MRI) [3]. While the radiotherapy regimens have been developed over the past decades from volumetric intensity-modulated arc therapy (VMAT) over intensity-modulated radiotherapy (IMRT) to possibly intensity-modulated proton therapy (IMPT) [4], there is an urgent need to specify the planning target volume of the tumour. The aim of improving the precision in tumour delineation of head and neck cancers is the reduction of the tumour recurrence rate and the minimization of side effects of the radiotherapy by sparing peritumoural normal tissue. Biological dose adaptation radiotherapy based on molecular and structural imaging has become an important field of research in the recent years [5, 6].

The standard tracer in oncology fluorine labelled fluordeoxyglucose ([^18^F]-FDG) has been evaluated with respect to the potential to deliver a valid prognosis based on the initial or interim PET/CT scans in a large number of studies [7]. These studies demonstrated a high predictive value of post therapy FDG uptake [8]. Radiotherapy planning studies using [^18^F]-FDG-PET/CT or [^18^F]-FDG-PET/MRI resulted in an improved detection of the gross tumour volume (GTV) in comparison with structural imaging (either CT or MRI) alone [9–11]. On the other hand, the limitations of [^18^F]-FDG-PET have to be considered when it comes to the specificity of the uptake since several structures in the neck show physiological or inflammation-related uptake of glucose influencing the correct contouring of the tumour significantly [9]. Another aspect that has to be taken into account is the value of interim imaging during treatment as an early treatment evaluation [12]. Also, in this setting, the lacking specificity of FDG with respect to the correlation to viable tumour cells has been reported.

Beyond metabolic imaging using FDG, also other molecular imaging biomarkers have been evaluated. The most promising ones being proliferation and hypoxia tracers. These tracers have been studied with regard to their potential to improve radiotherapy planning and evaluation of therapy success [5, 6].

The proliferation has been evaluated using radiolabelled thymidine analogue (FLT) [5, 6]. In general the studies pointed out a facilitation of therapy regimen selection and improved prediction of outcome in comparison with FDG [13]. The concept of detecting the tumour and defining tumour cells with radioresistance was evaluated implementing different hypoxia-delineating radiotracers, namely [^18^F]-FMISO, [^18^F]-FAZA and [^18^F]-HX4. For all three tracers, the tracer uptake correlated with the grade of hypoxia in the viable tumour cells [14]. Especially molecular imaging using the most promising hypoxia-related radiotracer [^18^F]-HX4, it effectively demonstrates changes in hypoxia by detecting the extent of hypoxic cells in the tumour volume during radiotherapy [15].

Unfortunately, all hypoxia tracers have one thing in common, they show only low to moderate tumour to background ratios and visualize only a small subpopulation of the tumour mass which disqualify the tracers for valuable radiotherapy planning [9].

In epithelial tumours, cancer-associated fibroblasts (CAFs) make up to 90% of the entire tumour volume, providing the base of the tumour stroma. They have been shown to facilitate cancer progression via supporting of the tumour cell growth extracellular matrix remodelling, angiogenesis promoting and mediation of tumour promoting inflammation [16]. One important protein CAFs express is the fibroblast activation protein (FAP). Recently gallium-68 labelled quinoline-based PET tracers that act as FAP inhibitors have been introduced ([^68^Ga]Ga-DOTA-FAPI) [17, 18].

[^68^Ga]Ga-DOTA-FAPI has shown excellent tumour to background ratios in oncologic imaging [17]. It is of note that a large variety of solid and non-solid tumours can be delineated using this innovative radiotracer [19]. Especially in head and neck cancers, ^68^Ga-DOTA-FAPI is very favourable because of its biodistribution with moderate to high tumour uptake and a low background activity in the surrounding tissue (especially normal lymphatic tissue like the tonsils) and the brain [18].

In this issue, Syed et al. evaluated the usefulness of implementing [^68^Ga]Ga-DOTA-FAPI PET data into the gross tumour volume delineation for targeted radiotherapy in head and neck cancers [20]. They compared conventional morphological radiation planning employing contrast enhanced CT with the performance of [^68^Ga]Ga-DOTA-FAPI in a total of 14 patients. FAPI displayed a high uptake within the tumour lesions (SUVmax 14.62 ± 4.44) and just a low background uptake within the salivary glands (SUVmax 1.76 ± 0.31). Using different SUV thresholds for automated contouring of the tumour lesions resulted in significantly larger median potentially to be irradiated volumes. Including all available clinical and imaging information a thresholding of three times the individual SUVmax of healthy tissue seemed to be reasonable. A merging of the CT and FAPI-based GTVs resulted in an approximately two times larger volume. The authors present first evidence of the value of FAP inhibitors in the biological based radiotherapy planning approach. However, next to a direct histopathological correlation, it is of large importance to also get information on the outcome of patients treated based on FAPI PET/CT data and by that get a confirmation of the assumed large potential of this new tracer.

Compared with earlier [18F]-FDG studies the results of the study using [^68^Ga]Ga-DOTA-FAPI seem to be very promising with respect to sensitivity and specificity. It is expected that especially the differentiation between tumour, physiological or inflammatory tracer uptake will be superior using [^68^Ga]Ga-DOTA-FAPI compared with the previously briefly discussed radiotracers [21].

As already shown by the recent ground-breaking studies using [^68^Ga]Ga-DOTA-FAPI in different tumour entities as a diagnostic tool, the current study gives an impressive insight into the potential of FAPI as a block buster in image-guided radiotherapy of head and neck cancers, where the well-established [^18^F]-FDG radiotracer has to face its limitations.

It is of great importance now that the nuclear medicine community will stand together and cooperate with radiation oncologists to accelerate the scientific research for [^68^Ga]Ga-DOTA-FAPI in radiotherapy planning, since this pioneering work by Syed et al. showed the immense potential of this radiotracer. Prospective trials are needed to outline a possible indispensability of [^68^Ga]Ga-DOTA-FAPI in the future of multimodal biological-guided head and neck cancer radiotherapy planning.
